# Immune Cell Profiling of the Cerebrospinal Fluid Provides Pathogenetic Insights Into Inflammatory Neuropathies

**DOI:** 10.3389/fimmu.2019.00515

**Published:** 2019-03-21

**Authors:** Michael Heming, Andreas Schulte-Mecklenbeck, Tobias Brix, Jolien Wolbert, Tillmann Ruland, Luisa Klotz, Sven G. Meuth, Catharina C. Gross, Heinz Wiendl, Gerd Meyer zu Hörste

**Affiliations:** ^1^Department of Neurology, Institute of Translational Neurology, University of Münster, Münster, Germany; ^2^Institute of Medical Informatics, University of Münster, Münster, Germany; ^3^Department of Psychiatry, University of Münster, Münster, Germany

**Keywords:** inflammatory neuropathies, Guillain-Barré syndrome, chronic inflammatory demyelinating polyneuropathy, immune cell profile, cerebrospinal fluid, flow cytometry

## Abstract

**Objective:** Utilize immune cell profiles in the cerebrospinal fluid (CSF) to advance the understanding and potentially support the diagnosis of inflammatory neuropathies.

**Methods:** We analyzed CSF cell flow cytometry data of patients with definite Guillain-Barré syndrome (GBS, *n* = 26) and chronic inflammatory demyelinating polyneuropathy (CIDP, *n* = 32) based on established diagnostic criteria in comparison to controls with relapsing-remitting multiple sclerosis (RRMS, *n* = 49) and idiopathic intracranial hypertension (IIH, *n* = 63).

**Results:** Flow cytometry revealed disease-specific changes of CSF cell composition with a significant increase of NKT cells and CD8+ T cells in CIDP, NK cells in GBS, and B cells and plasma cells in MS in comparison to IIH controls. Principal component analysis demonstrated distinct CSF immune cells pattern in inflammatory neuropathies vs. RRMS. Systematic receiver operator curve (ROC) analysis identified NKT cells as the best parameter to distinguish GBS from CIDP. Composite scores combing several of the CSF parameters differentiated inflammatory neuropathies from IIH and GBS from CIDP with high confidence. Applying a novel dimension reduction technique, we observed an intra-disease heterogeneity of inflammatory neuropathies.

**Conclusion:** Inflammatory neuropathies display disease- and subtype-specific alterations of CSF cell composition. The increase of NKT cells and CD8+ T cells in CIDP and NK cells in GBS, suggests a central role of cytotoxic cell types in inflammatory neuropathies varying between acute and chronic subtypes. Composite scores constructed from multi-dimensional CSF parameters establish potential novel diagnostic tools. Intra-disease heterogeneity suggests distinct disease mechanisms in subgroups of inflammatory neuropathies.

## Introduction

Guillain-Barré syndrome (GBS) and chronic inflammatory demyelinating polyneuropathy (CIDP) are the most frequent of the heterogeneous group of immune-mediated neuropathies. Both can cause considerable and often permanent disability (1). The diagnosis of GBS and CIDP can remain challenging despite the existence of diagnostic criteria for GBS (2) and CIDP (3). This is in part due to (1) atypical clinical presentations (4), (2) technical difficulties in nerve conduction studies (5), and (3) low specificity of diagnostic criteria (6). Diagnosis is further impeded by the overlap between recurrent GBS and relapsing CIDP courses. Therefore, better mechanistically understanding and distinguishing the heterogeneity of inflammatory neuropathies would be of considerable clinical relevance, especially in the light of differential treatment requirements (7).

In contrast to the vast expansion of knowledge in CNS autoimmunity, comparably little progress has been made in the understanding of PNS autoimmunity; especially regarding the immunological factors differentiating acute from chronic immune neuropathies. Using CSF, some of the few available studies found a specific cytokine profile in the CSF of CIDP patients (8, 9). Other studies reported changes of Th17 cells (10) and NK cells (11). Certain T helper cell populations are elevated in the CSF in GBS (12) and in a corresponding animal model (13). Despite these scattered observations, a comprehensive immune cell profiling of the CSF has not been performed.

We here performed a systematic retrospective analysis of flow cytometry profiling of CSF leukocytes, in patients with definite GBS and CIDP in comparison to relapsing-remitting multiple sclerosis (RRMS) and idiopathic intracranial hypertension (IIH) to aid the understanding and diagnosis of inflammatory neuropathies.

## Methods

### Patients

In our center, all CSF samples obtained during regular working hours are routinely analyzed by flow cytometry. We retrospectively searched files of patients who had been admitted to the Department of Neurology at the University Hospital Münster between years 2012 and 2018 for the ICD-10 diagnosis codes G61.0, G61.8, G35.1, and G93.2 and who had received lumbar puncture (LP) for routine CSF analysis and CSF cell flow cytometry. In total, we identified 26 patients with GBS, 32 patients with CIDP, 49 patients with RRMS, serving as a reference group with known inflammatory CSF changes, and 63 patients with IIH, serving as a control group with mostly normal CSF (14). Only GBS patients who fulfilled Brighton criteria (2) level 1 were admitted to the study. Only CIDP patients who had a definite CIDP according to the EFNS/PNS diagnostic criteria (3) were included in the study. In addition, Hughes disability score (0—healthy, 1—minor signs of neuropathy, but capable of manual work, 2—able to walk with aid of stick, but incapable of manual work, 3—able to walk with support, 4—confined to bed or chairbound, 5—requiring assisted ventilation, 6—death) (15) and the modified Rankin scale (mRS) were determined on day of admission. For the disease severity analysis the mRS was dichotomized to classify mildly (mRS 1–2) and severely (mRS 3–6) affected patients. Patients with RRMS were selected according to the 2017 revision of the McDonald criteria (16). Patients who had been treated with alemtuzumab, ocrelizumab, cladribine, or rituximab in the last year or with fingolimod, natalizumab, mitoxantrone, cyclophosphamide, methotrexate, ciclosporin A in the last 3 months were excluded from all cohorts. Current treatments with interferons, glatiramer acetate, dimethyl fumarate, azathioprine, steroids, or intravenous immunoglobulins were accepted.

### CSF Flow Cytometry

LPs were performed under sterile conditions using 20G Sprotte Canulae (Pajunk Medical). All samples were pseudonymized at collection. CSF was transported to further processing as quickly as possible and centrifuged at 300 g for 15 min. The supernatant was removed and CSF cells were stained for flow cytometry as described previously (17–19). Briefly, CSF flow cytometry was performed using a Navious flow cytometer (Beckman Coulter). Cells were incubated in VersaLyse buffer and stained using the following anti-human antibodies (Beckman Coulter; clone names indicated): CD3 (UCHT1); CD4 (13B8.2); CD8 (B9.11); CD14 (RMO52); CD16 (3G8); CD19 (J3-119); CD45 (J.33); CD56 (C218); CD138 (B-A38); HLA-DR (Immu-357). Gating was first by forward scatter (FSC)/sideward scatter (SSC) and subsequently on CD45+ cells and the percentage of cell populations was assessed for further analysis. In detail, cell populations were defined as follows: *CD45 cells*: %CD45+ of all events, l*ymphocytes*: % of cells gated as lymphocytes by FSC/SSC of CD45+ cells, *monocytes*: % of cells gated as monocytes by FSC/SSC of CD45+ cells, *T cells*: %CD3+CD56- of lymphocytes, *CD4 cells*: %CD4+ of T cells, *CD8 cells*: %CD8+ of T cells, *CD4CD8 cells*: %CD4+CD8+ of T cells, *CD4/CD8 ratio*: %CD4+ of T cells / %CD8+ of T cells, *HLA-DR T cells*: %HLA-DR+ of T cells, *HLA-DR CD4 T cells*: %HLA-DR+ of CD4 cells, *HLA-DR CD8 T cells*: %HLA-DR+ of CD8 cells, *NK cells*: %CD56+CD3- of lymphocytes, *NKT cells*: %CD56+CD3+ of lymphocytes, *HLA-DR NK cells*: %HLA-DR+ of NK cells, *CD56dim CD16*+ *NK cells*: %CD56dimCD16+ of NK cells, *CD56bright CD16- NK cells*: %CD56brightCD16- of NK cells, *B cells*: %CD19+ of lymphocytes, *plasma cells*: %CD138+ of lymphocytes, *classical monocytes*: %CD14+CD16-/dim of monocytes, *non-classical monocytes*: %CD14+/lowCD16+ of monocytes (Supplementary Figure 1). CSF protein concentration, albumin, IgG, IgA, and IgM levels in the CSF were analyzed using nephelometry. A Reiber scheme was created for each Ig and we evaluated the presence of a BBB disruption or an intrathecal Ig synthesis. We used isoelectric focusing followed by silver nitrate staining to detect oligoclonal bands (OCBs). Of note, OCBs, BBB disruption, and intrathecal Ig synthesis are dichotomous parameters, while all other parameters are continuous.

### Statistical Analysis

Statistical analysis of the data was performed using *R* version 3.5.1. The statistical significance of the data was determined using either the chi-squared test for comparing frequencies, the Mann-Whitney U-test for comparing two groups or the Kruskal-Wallis test with the Dunn test as a *post hoc* test when performing multiple comparisons. Correction for multiple testing was performed by Benjamini-Hochberg's false discovery rate correction. A *p* < 0.05 was considered statistically significant. Clustered heatmaps were created with the R package pheatmap. First of all, the mean of each parameter was calculated categorized by disease. To improve comparability, the results were scaled and centered by subtracting the column means from their corresponding column and dividing the columns by their standard deviations. Hierarchical clustering of rows was performed with complete linkage clustering and Euclidean distance measure. Correlation matrix was calculated with Spearman's rank correlation coefficient and data were hierarchically clustered with complete linkage and Euclidean distance measure. To reduce dataset dimensionality and detect patters of CSF data, principal component analysis (PCA) was performed with the R package factoextra treating each patient as one datapoint. Furthermore, to visualize our complex data we used a recently published dimension reduction technique, the uniform manifold approximation and projection for dimension reduction (UMAP) (20), which represents a further development of the t-Distributed stochastic neighbor embedding algorithm (t-SNE) (21). To investigate the most suitable parameters for distinguishing between patients with CIDP, GBS, RRMS, and IIH, receiver operating characteristics (ROC) analysis was performed with the R package pROC (22). A ROC analysis allows systematically evaluating the sensitivity and specificity of a test and returns area under the curve (AUC) values. An AUC of 0.5 represents an uninformative classifier, while an AUC of 1 indicates perfect performance (23). When multiple predictors were used for ROC analysis, we performed a generalized linear model with logistic regression by adding multiple parameters in advance. The optimal number of parameters was determined by the Bayesian information criteria. The composite scores were selected by the regsubsets function of the R-package leaps using exhaustive search. The 95% confidence interval was calculated using De Long test.

### Standard Protocol Approvals, Registrations, and Patient Consents

The study was conducted according to the declaration of Helsinki and approved by the local ethical committee (AZ 2018-563-f-S).

## Results

### Patient Characteristics and Validation of the Approach

**Figure 1:**
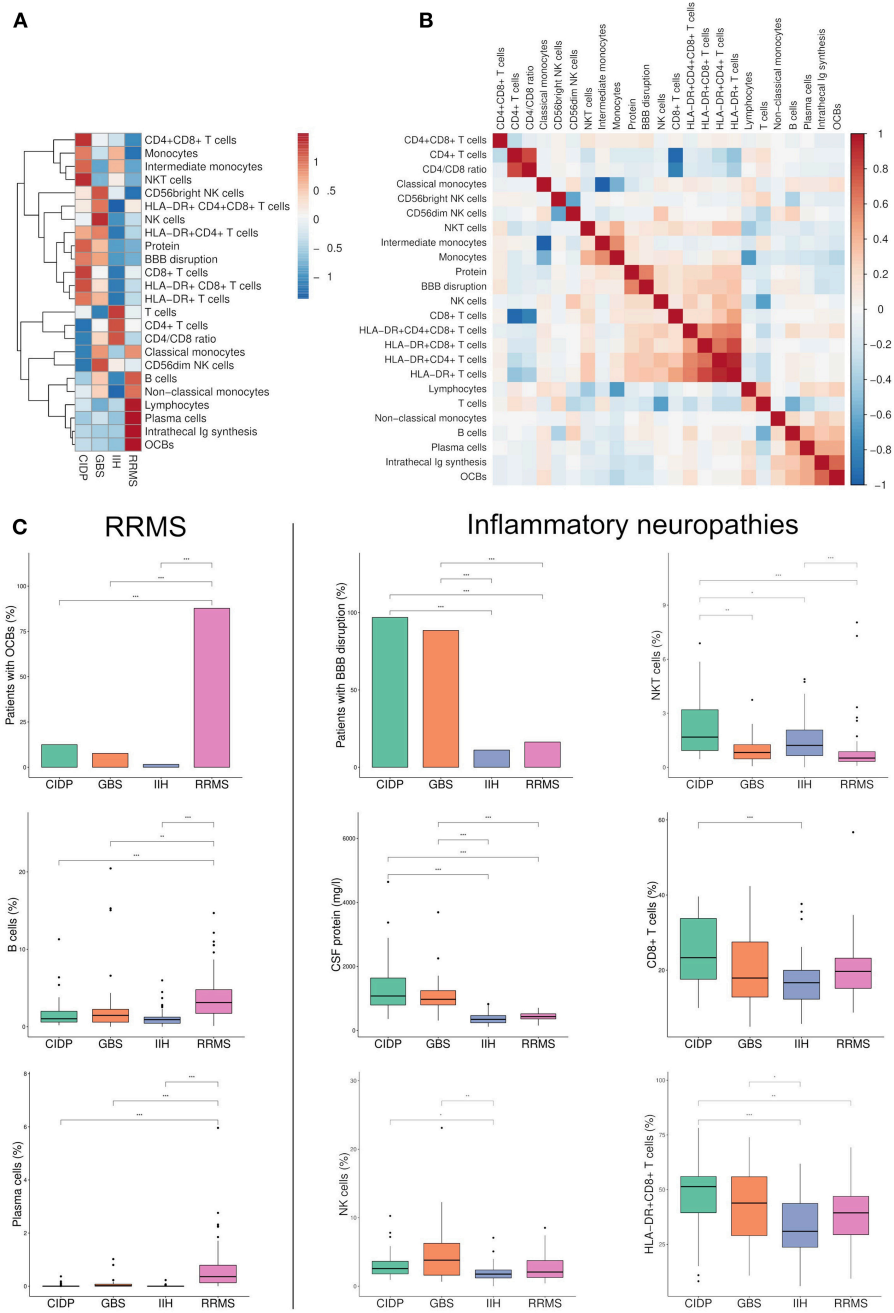
Flow cytometry identifies new parameters to discriminate between immune-mediated neuropathies. **(A)** Heatmap depicting row mean of each CSF parameter per row calculated for chronic inflammatory demyelinating neuropathy (CIDP), Guillain-Barré syndrome (GBS), relapsing-remitting multiple sclerosis (RRMS) and idiopathic intracranial hypertension (IIH). The means were scaled and centered for each row by subtracting the column means from their corresponding column and dividing the columns by their standard deviations. Next, hierarchical clustering was performed with complete linkage method and Euclidean distance measure and visualized in a heatmap. **(B)** A correlation matrix of the investigated parameters was calculated with Spearman's rank correlation coefficient. Correlations coefficients were clustered hierarchically with the single linkage method and Euclidean distance measure. The correlation coefficients are colored according to the value. Positive correlations are displayed in red, negative correlations are colored in blue. **(C)** Box plots and bar plots of selected CSF parameters categorized by diagnosis. RRMS-related markers are shown on the left, markers related to inflammatory neuropathies are displayed on the right. Boxes indicate the lower quartile, median, and upper quartile with whiskers extending to the furthest value within 1.5 times the interquartile range of the box. Outliers are identified individually. The statistical significance of the results was determined using Kruskal-Wallis test and the Dunn test as a *post hoc* test. Correction for multiple testing was performed by Benjamini-Hochberg's false discovery rate correction. ^*^p < 0.05, ^**^p < 0.01, ^***^p < 0.001. BBB, blood-brain barrier; OCBs, oligoclonal bands; Ig, immunoglobulin.

First, we characterized the patient cohorts. Patients with IIH and RRMS were younger and more often female than patients with GBS and CIDP and the lag between onset of symptoms in GBS was shorter than in CIDP (Table 1) (24–27). The percentage of non-treated patients in CIDP and GBS was comparable (Supplementary Figure 3, Table 1). The most common therapy in both groups was intravenous immunoglobulins (Supplementary Figure 3, Table 1). We identified 4 out of 32 CIDP patients that were initially misdiagnosed as GBS because of a rapid-onset with consecutive chronic course. All were later correctly classified as CIDP patients (Table 1). As expected, RRMS patients showed mildly elevated cell counts in CSF as well as increased proportions of intrathecal immunoglobulin (Ig) synthesis, and presence of oligoclonal bands (OCBs) (Figures 1A,C) (28). In contrast, CSF protein and blood-brain barrier (BBB) disruption were significantly increased in GBS and CIDP patients (Figure 1C).

**Table 1:** Demographics and basic CSF characteristics of the patients.

	**CIDP**	**GBS**	**IIH**	**RRMS**
Number of patients	32 (4 A-CIDP)	26	63	49
Age (median with range)	58 (18–78)	59 (18–83)	32 (18–76)	33 (18–55)
Female (number/percent)	6/18.7%	12/46.2%	50/79.4%	33/67.3%
Male (number/percent)	26/81.3%	14/53.8%	13/20.6%	16/32.7%
CSF cells (median with range) /μl	1 (0–12)	1 (0–37)	1 (0–5)	4 (0–58)
CSF protein (median with range) mg/l	1075 (358–4640)	972 (308–3690)	345 (115–823)	431 (152–705)
BBBD (number/percent)	31/96.8%	23/88.5%	7/11.1%	8/16.3%
Intrathecal Ig synthesis (number/percent)	1/1.6%	0/0 %	1/1.6%	30/61.2%
OCBs (number/percent)	4/12.5%	2/7.7%	1/1.6%	43/87.8%
Hughes Score (median with range)	2 (1–4)^**^ Mean: 2.1	3 (1–5)^**^ Mean: 3.1		
Modified Rankin Scale (median with range)	3 (1–4)^*^ Mean: 2.5	4 (1–5)^*^ Mean: 3.6		
Non-treated patients in the last 3 months (number/percent)	18/56.2%	15/57.7%		
Therapy with IVIGs in the last 3 months (number/percent of treated patients)	8/57.1%	6/54.5%		
Time between onset of symptoms and sampling (median) days	485	10		

A-CIDP, Acute onset CIDP; BBBD, blood-brain barrier disruption; CIDP, chronic inflammatory demyelinating polyneuropathy; GBS, Guillain-Barré syndrome; IIH, idiopathic intracranial hypertension; Ig, immunoglobulin; OCB, oligoclonal band; IVIG, intravenous immunoglobulin; RRMS, relapsing remitting multiple sclerosis

* *p < 0.05*,

** *p < 0.01 (calculated by Mann-Whitney U-test)*.

We next collected and systematically analyzed multi-dimensional flow cytometry data of CSF cells that are routinely obtained in our center together with standard CSF parameters. Supplementary Figure 2 displays representative flow cytometry data of each diagnosis. As described (29), CSF in RRMS patients showed an expansion of plasma cells and B cells and increased frequencies of OCBs and intrathecal Ig synthesis as indicators of intrathecal monoclonal B cell responses (Figures 1A,B). Classical monocytes and non-classical monocytes, which were recently shown to play a pivotal role in the pathophysiology of MS (30), were also expanded in MS. This served as a positive control that our approach indeed replicates known inflammatory changes.

### Disease- and Subtype-Specific CSF Alterations in Inflammatory Neuropathies

In GBS and CIDP, CSF exhibited known disease-associated changes including increased protein concentration and BBB disruption (i.e., “cytoalbuminologic dissociation”), with no differences between GBS and CIDP (Figures 1A,C). Non-specific indicators of lymphocyte activation (i.e., HLA-DR+ T cell populations) were also increased in line with the inflammatory etiology of both diseases. Similar changes have been previously described in various neurological diseases (17, 31). Notably, CSF of GBS patients displayed a disease-specific increase of NK cells (Figures 1A,C). The CSF of CIDP patients showed some overlapping, but also distinct changes and featured an increase of NKT cells and CD8+ T cells (Figures 1A,C). Inflammatory neuropathies thus exhibit a disease- and subtype-specific pattern of CSF cell abnormalities indicating a relevance of cytotoxic immune responses in both diseases that is distinct between subtypes.

We next aimed to understand the interdependence of individual CSF parameters and therefore performed a correlation analysis (Figure 1B). We found that some parameters formed two distinct and apparently co-regulated modules while the remaining parameters showed no apparent inter-relation. One module was best described as representing leukocyte activation (i.e., HLA-DR+ populations) and more widely included elevation of CNS protein, BBB disruption, NK cells, CD8+ T cells, and NKT cells and could thus be assigned to inflammatory neuropathies. The second module was best described as B cell-related (e.g., B cells, plasma cells, OCB, intrathecal immunoglobulin synthesis) and therefore matched the immune cell profile of RRMS (Figure 1B). As expected, given their reciprocal relationship, the proportion of CD4+ and CD8+ T cells and classical and non-classical monocytes were each negatively correlated. CSF parameters thus form mechanistically related clusters.

### Disability Increases With CSF Indicators of Inflammation

We sought to find CSF parameters that correlate with the severity of the disease, as defined by the Hughes disability score (HDS) and the modified Rankin scale (mRS). Interestingly, there was a significant positive correlation between HLA-DR+CD4+ cells and HDS/mRS (Spearman correlation coefficient = 0.4/0.42, *p* = 0.041/0.033) and between non-classical monocytes and HDS/mRS (Spearman correlation coefficient = 0.51/0.49, *p* = 0.0075/0.01) in GBS patients (Supplementary Figure 6A). In CIDP patients, we detected a significant positive correlation between CSF protein concentration and HDS/mRS (Spearman correlation coefficient = 0.37/0.39, *p* = 0.039/0.027) (Supplementary Figure 6B). This indicates that markers of inflammation positively correlate with disease severity.

### Distinct Disease-Specific Immune Cell Profiles

**Figure 2:**
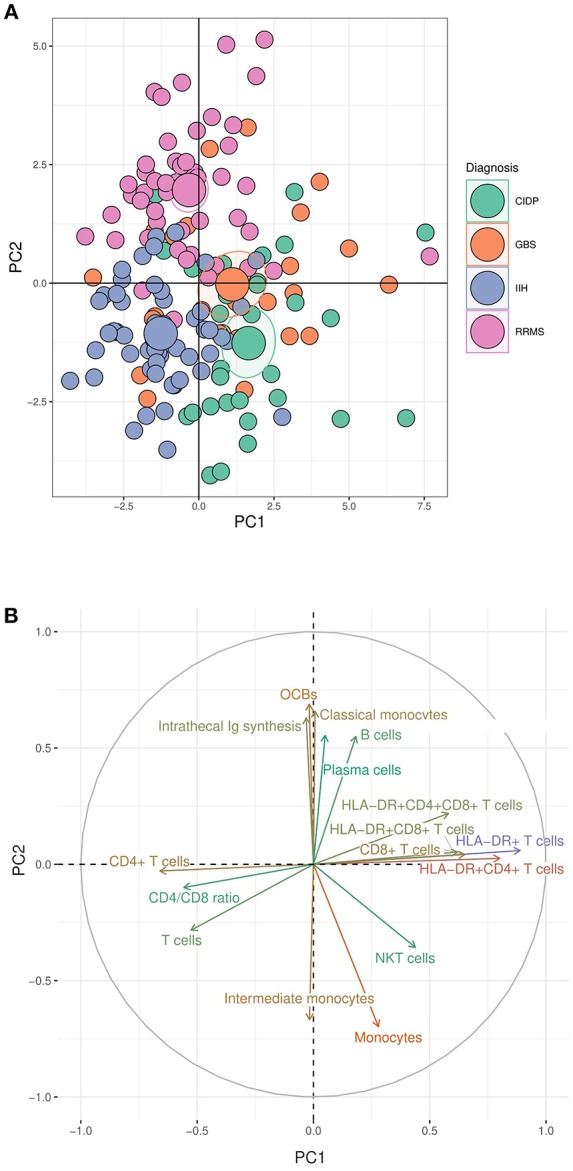
Dimensionality reduction reveals distinct immune cell profiles of inflammatory neuropathies. **(A)** Principal component (PC) analysis (PCA) was performed using data from patients with chronic inflammatory demyelinating polyneuropathy (CIDP), Guillain-Barré syndrome (GBS), relapsing-remitting multiple sclerosis (RRMS), and idiopathic intracranial hypertension (IIH). Each circle represents a single patient; larger circles represent the group mean. The ellipses around each group mean point represent the confidence intervals. **(B)** The top 16 parameters contributing to PC1 and PC2 in the PCA are depicted. A long distance between a variable and the origin indicates a major influence of the corresponding variable on the PC. OCBs, oligoclonal bands; Ig, immunoglobulin.

To aid the understanding of our complex dataset, we performed principal component analysis (PCA) regarding patients as multi-dimensional data points (Figure 2A). PCA illustrated that RRMS, IIH and CIDP/GBS patients each formed distinct clusters of CSF profiles, which were significantly different with non-overlapping confidence interval (Figure 2A). In contrast, CIDP and GBS showed some overlap. Next, we aimed to understand which parameters controlled PCA clustering of diseases. Principal component (PC) 2 determined the difference between RRMS vs. IIH, with the main contributors being parameters of the B cell lineage (plasma cells, B cells, OCBs, and intrathecal Ig synthesis) (Figure 2B). In contrast, PC1 determined the difference between GBS/CIDP vs. IIH and its main contributors were activated and non-activated CD8+ T cells, as well as NKT cells (Figure 2B). The main loadings of PC1 and PC2 thus corresponded well to known disease-specific alterations in MS and again supported T/NK cell-driven pathology in GBS/CIDP. This illustrates the applicability of dimensionality reduction techniques to understanding clinical datasets.

### Differentiating Inflammatory Neuropathies With CSF Parameters

**Figure 3:**
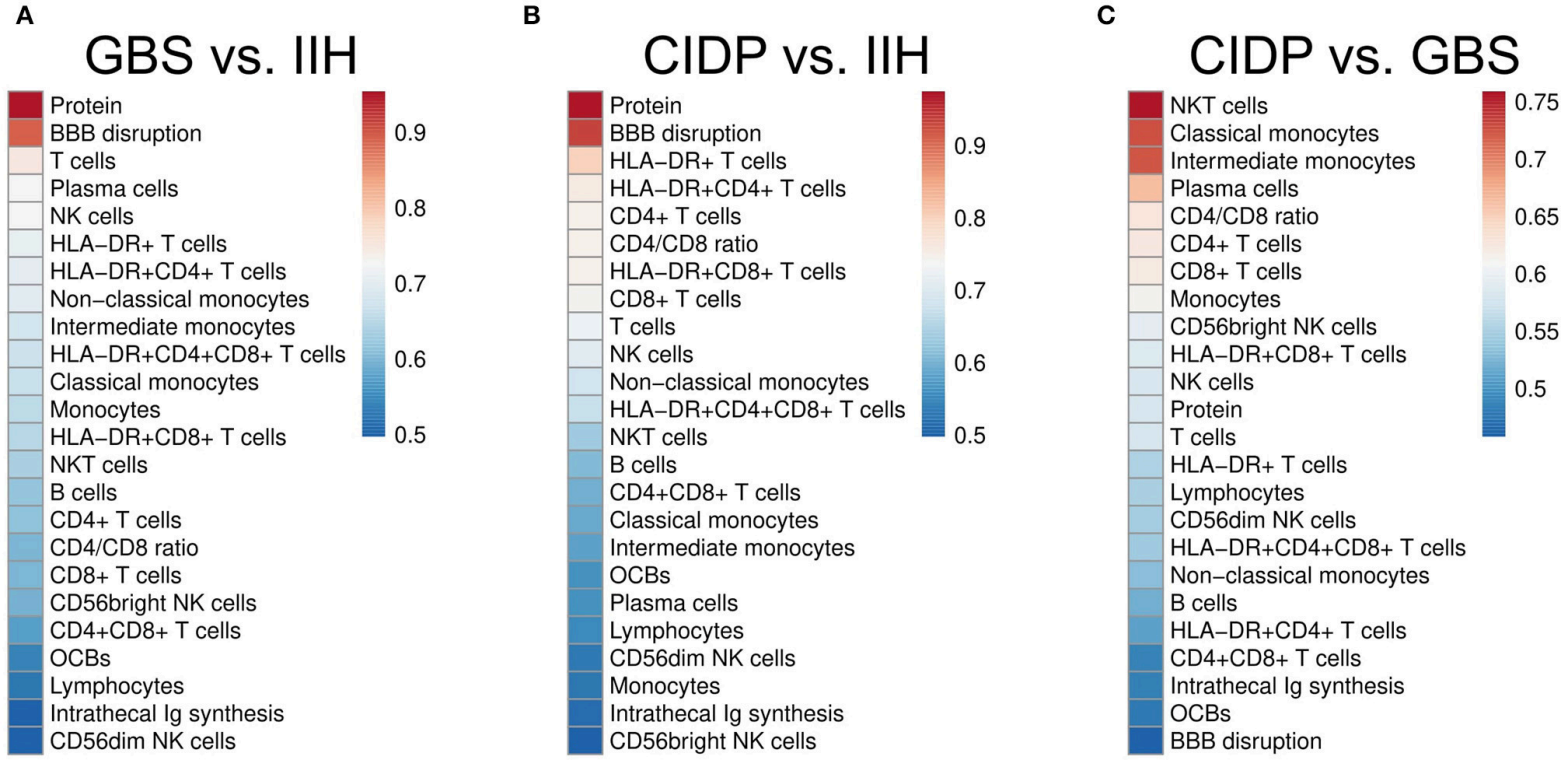
CSF parameters differentiate inflammatory neuropathies with good confidence. Receiver operating characteristics (ROC) analysis was performed for each CSF parameter in different subsets. Heatmaps depict the color-coded values of the area under the curve (AUC) of the ROC analyses of different subsets sorted by size. **(A)** Guillain–Barré syndrome (GBS) vs. idiopathic intracranial hypertension (IIH). **(B)** Chronic inflammatory demyelinating neuropathy (CIDP) vs. IIH. **(C)** CIDP vs. GBS. BBB, blood-brain barrier; OCBs, oligoclonal bands; Ig, immunoglobulin. Please note the different color scales.

Next, we sought to systematically test our findings for potential diagnostic value. The area under the curve (AUC) in a ROC analysis can be used to measure the quality of a diagnostic test ranging from acceptable (AUC 0.7–0.8) through excellent (AUC 0.8–0.9) to outstanding (AUC >0.9) (32). We thereby tested which individual CSF parameter best differentiated inflammatory neuropathies from controls and GBS from CIDP. As expected, CSF protein (AUC 0.95) and BBB disruption (AUC 0.894) distinguished GBS from IIH patients, followed by activated and non-activated T cells, but also NK cells (AUC 0.73) (Figure 3A, Supplementary Table 1). The parameters discriminating CIDP from IIH were again CSF protein (AUC 0.97) and BBB disruption (AUC 0.94) and activated and non-activated T cells (Figure 3B, Supplementary Table 2).

Next, we searched for parameters distinguishing GBS from CIDP and found that NKT cells were the best parameter (AUC 0.76) differentiating CIDP and GBS followed by classical and intermediate monocytes (Figure 3C, Supplementary Table 3). CSF protein concentration and BBB disruption did not discriminate well between CIDP and GBS since they were elevated in both diseases. We thus identify a first potential surrogate parameter distinguishing CIDP from GBS—a clinically relevant, but difficult differentiation.

### Composite Scores of CSF Parameters

**Figure 4:**
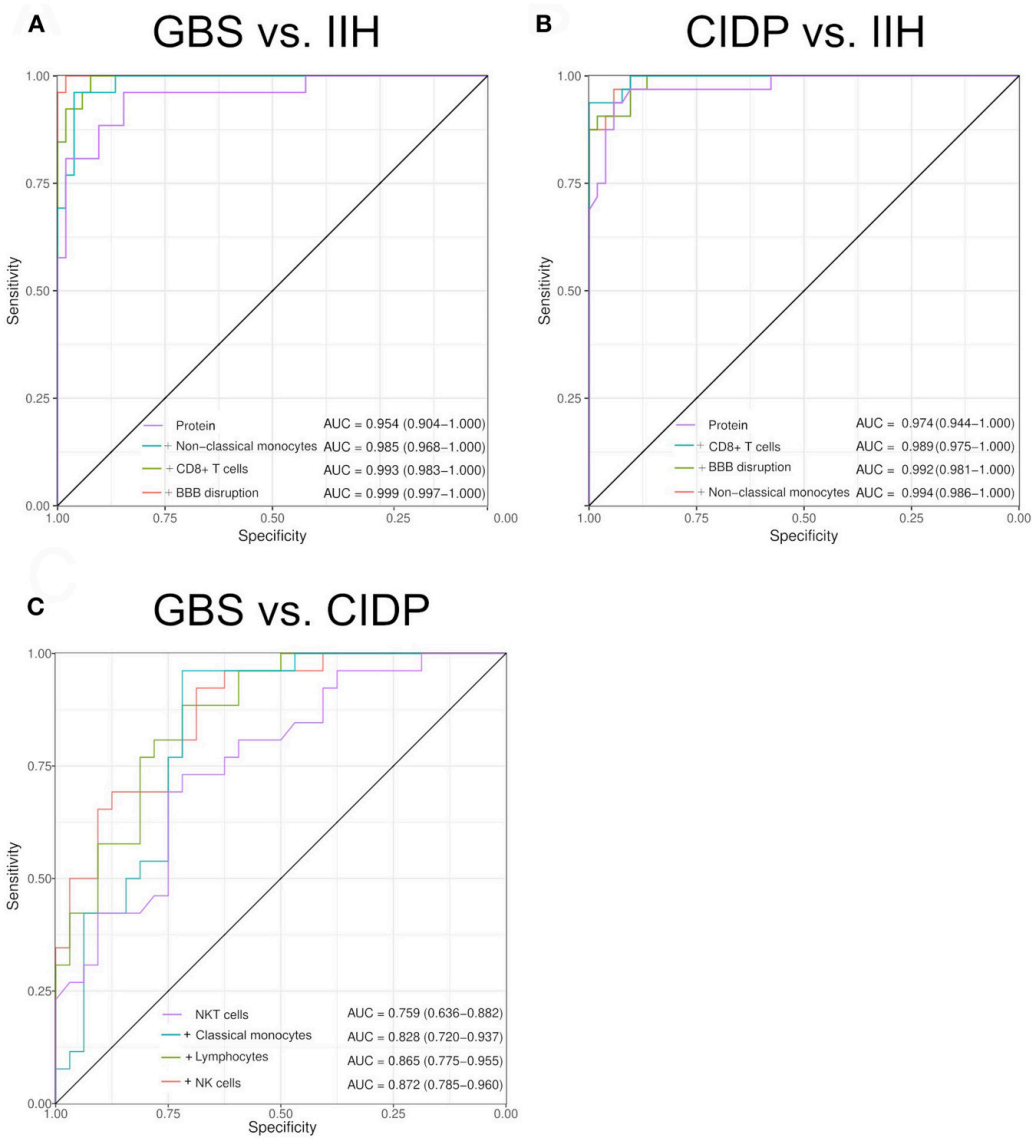
Composite scores of multiple CSF parameters allow differentiating inflammatory neuropathies with high sensitivity and specificity. Two to five CSF parameters were combined by a generalized linear model with logistic regression. Receiver operating characteristics (ROC) analysis was performed. We increased the number of parameters sequentially. The model selection was performed by an exhaustive search. The maximal number of parameters was determined by the Bayesian information criteria. The individual parameters that form the composite score and the resulting AUC are given in the respective figure. The confidence was calculated using De Long test and is indicated in brackets. **(A)** Guillain–Barré syndrome (GBS) vs. idiopathic intracranial hypertension (IIH). **(B)** Chronic inflammatory demyelinating neuropathy (CIDP) vs. IIH. **(C)** CIDP vs. GBS. BBB, blood-brain barrier.

We then speculated that combining individual parameters could improve the discriminatory ability of multi-parametric CSF analysis and therefore calculated summed composite scores. Models were determined computationally with the maximal number of parameters limited to four (see Methods for details). These summed composite scores improved diagnostic accuracy slightly for the comparisons of GBS against IIH (composite AUC 0.999 vs. single AUC 0.954) (Figure 4A) and for CIDP against IIH (composite AUC 0.994 vs. single AUC 0.974) (Figure 4B). Overall, the diagnosis of inflammatory neuropathies thus mostly relied on CSF protein and additional parameters provided minor benefit. However, the differentiation between CIDP and GBS was different. Adding classical monocytes, T cells, activated NK cells and intrathecal Ig synthesis improved the AUC from 0.759 to 0.872 (Figure 4C). Flow cytometry-based composite scores might thus help differentiating subtypes of inflammatory neuropathies in the future.

### Intra-Disease Heterogeneity of Inflammatory Neuropathies

**Figure 5:**
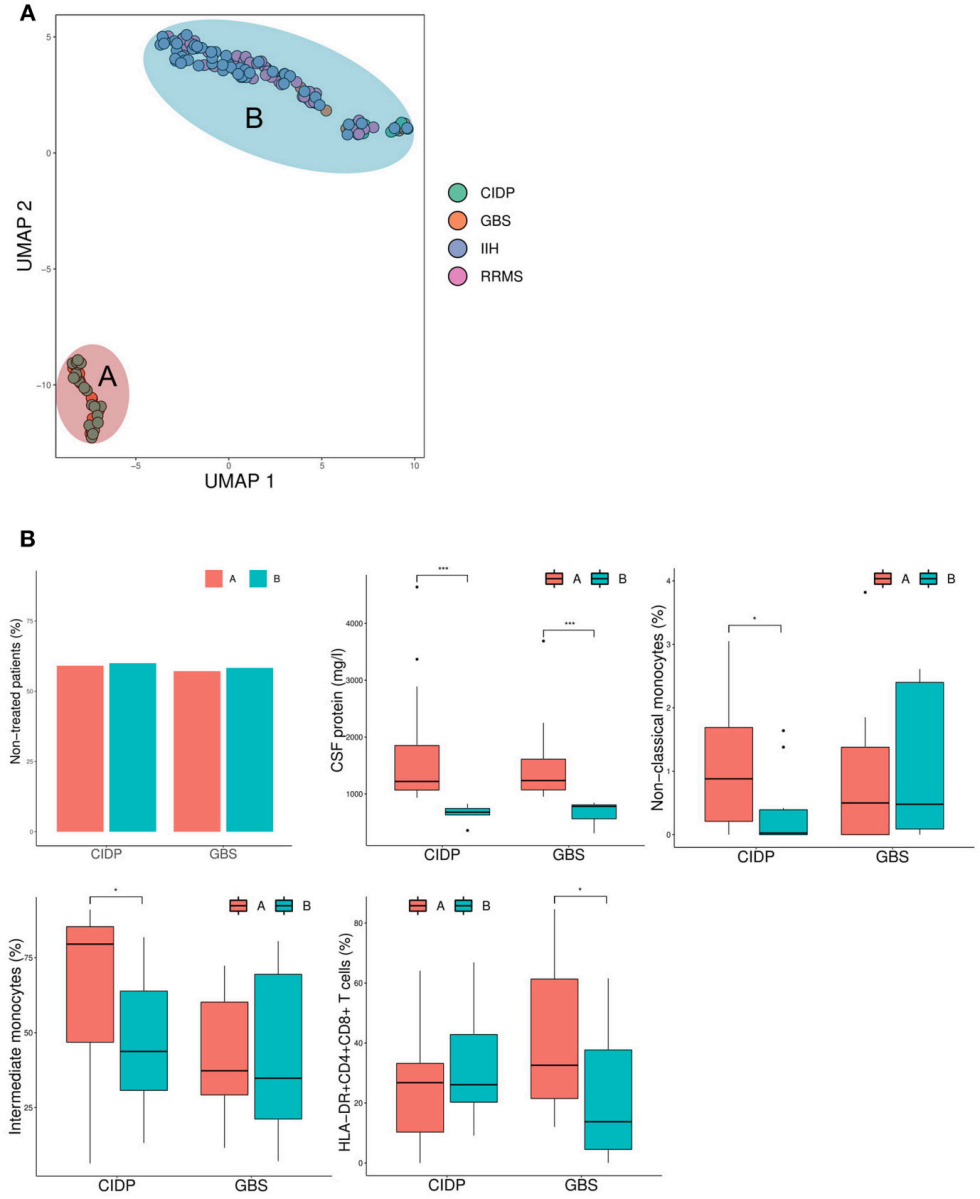
Inflammatory neuropathies show intra-disease heterogeneity characterized by CSF protein and monocytes. **(A)** The uniform manifold approximation and projection for dimension reduction (UMAP) (20), a recently published dimension reduction technique, was performed. Each circle represents a single patient and diagnosis is color coded. Based on the results of UMAP, patients were divided into group A and B as denoted. **(B)** Box plots show the CSF parameters that were significantly different between group A and group B. Boxes indicate the lower quartile, median, and upper quartile with whiskers extending to the furthest value within 1.5 times the interquartile range of the box. Outliers are identified individually. The statistical significance of the results was determined using the Mann-Whitney U-test for continuous variables (CSF protein, non-classical monocytes, intermediate monocytes, HLA-DR+CD4+CD8+ T cells) and the chi-squared test for the dichotomous variable (therapy). ^*^p < 0.05, ^***^p < 0.001. CIDP, chronic inflammatory demyelinating neuropathy; GBS, Guillain-Barré syndrome; RRMS, relapsing-remitting multiple sclerosis; IIH, idiopathic intracranial hypertension.

To further detect disease-specific CSF patterns, we performed a dimensionality reduction technique named uniform manifold approximation and projection for dimension reduction (UMAP) (20). In contrast to PCA, UMAP is non-linear and therefore less prone to outliers. This analysis identified two apparent subgroups of patients (Figure 5A) and thereby a surprising intra-disease heterogeneity of immune-mediated neuropathies. We classified inflammatory neuropathy patients based on the UMAP plot into group A, which formed a homogeneous cluster of CIDP and GBS patients, and group B, which showed considerable overlap with RRMS and IIH patients (Figure 5A). In comparison to group B, all patients from group A showed an elevated CSF protein concentration. Furthermore, group A displayed an increase of non-classical and intermediate monocytes in CIDP and an elevation of HLA-DR+CD4+CD8+ T cells in GBS (Figure 5B). Of note, this segregation was not driven by diagnostic certainty or treatment as the proportion of therapy-naïve patients was very similar in CIDP (59.1 % group A vs. 60% group B; chi-squared *p* = 1) and in GBS patients (57.1% group A vs. 58.3% group B; chi-squared *p* = 0.98) (Figure 5B). Based on these CSF surrogates, inflammatory neuropathy patients thus subset into two groups characterized by high CSF protein and raised non-classical monocytes in CIDP and elevated activated double positive T cells in GBS (group A) vs. low protein and overlap with control patients (group B). These findings suggest distinct disease mechanisms in subgroups of inflammatory neuropathies.

## Discussion

We here use multi-parametric CSF analysis combined with novel analytical approaches to identify disease- and subtype-specific changes in inflammatory neuropathies. Both GBS and CIDP show activation and elevation of cytotoxic immune cells in the CSF compartment that will form the basis for future mechanistic studies. In contrast, multiple sclerosis exhibits known signs of intrathecal B cell responses. The elevation of NKT cells and CD8+ T cells in CIDP and NK cells in GBS suggests a pivotal role of these cytotoxic cells in the pathophysiology of inflammatory neuropathies and could constitute a novel therapeutic target. We systematically evaluate these newly identified parameters for diagnostic value and identify NKT cells as a first potential surrogate parameter, distinguishing GBS from CIDP with moderate accuracy. Constructing a novel composite score further improved the ability to distinguish GBS form CIDP. Our findings thus suggest diagnostic options in inflammatory neuropathies by immune cell profiling of the CSF in the future.

The understanding of the heterogeneity and pathophysiology of PNS autoimmunity remains limited. Our observations of elevated CD8+ T cells is in line with recent studies, which suggested a key role of CD8+ T cells in CIDP (33, 34). In GBS, increased levels of T cells, NK cells and macrophages were found in peripheral nerves of an animal GBS model (13). Accordingly, we now found elevated levels of NK cells and activated T cells in GBS suggesting that peripheral nerve and other immune compartments may communicate. At first glance, treated GBS patients differ in their immune cell profile compared to non-treated GBS patients (Supplementary Figure 4). However, the broad NK signal in the full cohort becomes more restricted to the CD56bright subset of NK cells in untreated GBS patients suggesting a more specific subset expansion. In addition, treatment and disease severity are mutual confounders as severely affected patients are more likely to be treated (Supplementary Figure 5B). In fact, immune cell profiles of severely affected GBS patients were similar to treated GBS patients (Supplementary Figures 5A,C). We thus speculate that differences between treated and non-treated GBS patients are mainly due to disease severity. We observed positive correlations between GBS disability and NK cells and activated T cells highlighting their important pathophysiological role. We are the first to observe expansion of NKT cells specifically in CIDP, but not in GBS. Of note, this cell immune profile was equally observed in treated and non-treated CIDP patients indicating that the observed immune cell pattern characterizes CIDP irrespective of previous treatments. NKT cells represent an innate-like T cell subset that express an invariant chain of the T cell receptor and recognize peptide antigens by CD1d (35). Depending on the tissue, NKT cells play either protective (36) or pathological roles (37) in various diseases. For example, NKT cells were described to protect mice from EAE, the mouse model of MS (38). NKT are usually subdivided in classical and non-classical NKT cells that probably represent functionally distinct cells types (39). A deeper analysis of NKT cells may thus reveal a more detailed understanding of the pathogenetic role of NKT cells in inflammatory neuropathies. The addition of humoral markers, such as sCD21 (40), sCD27 (40), TACI (41), and YKL-40 (42), may help to distinguish MS subtypes and such proteomics-based approaches of CSF could also be relevant for identifying novel mechanisms in inflammatory neuropathies. Further research on the functional role of NKT cells in CIDP will be required to potentially identify a promising target for immune therapy (35).

Despite well-defined diagnostic criteria, immune-mediated neuropathies are often misdiagnosed. In a recent study, the reliance on subjective perception and liberal electrophysiologic interpretation were identified as common diagnostic errors (43). Therefore, objective parameters to correctly diagnose immune-mediated neuropathies are of high importance. Especially, differentiating GBS from CIDP is of high clinical relevance since treatment options are different (44) and a first episode of relapsing CIDP may easily be confused with GBS initially (45, 46).

Our study has clear limitations due to its retrospective study design and comparably small patient cohort. However, recruiting rare inflammatory neuropathy patients for an analytical method that requires fragile CSF cells to be analyzed immediately with extensive technical equipment is very challenging in a prospective study design. To the best of our knowledge, our study therefore constitutes the first comprehensive flow cytometry characterization of CSF cells in inflammatory neuropathies and articulates new mechanistic hypotheses. It will be important to evaluate our single parameters and composite scores evaluated here against other neuropathy controls in a more real-world clinical scenario with higher sample sizes. Hereafter, scores could help diagnosing inflammatory neuropathies in the future.

Furthermore, our data suggest a previously unknown intra-disease heterogeneity of inflammatory neuropathies that is driven by CSF protein, specific players of innate immunity in CIDP and activated T cells in GBS. It is tempting to speculate that these different groups differ in pathophysiology and may require different therapeutic approaches.

In summary, our cellular immune profiling flow cytometry of CSF cells adds to the understanding of divergent pathogenetic traits between GBS and CIDP paving the way for subsequent mechanistic studies. Furthermore, our composites scores represent potential tools in the diagnosis of immune-mediated neuropathies that are objective and can be easily standardized.

## Data Availability

The datasets generated for this study are available on request to the corresponding author.

## Author Contributions

MH performed data acquisition and data analysis with statistical analysis and drafted the manuscript. AS-M, TB, JW, and TR contributed to data acquisition and analysis. SM, LK, CG, and HW revised the manuscript and co-supervised the project. GMzH conceptualized the project, revised the manuscript, and supervised the project.

### Conflict of Interest Statement

LK received compensation for serving on Scientific Advisory Boards for Genzyme and Novartis; received speaker honoraria and travel support from Novartis, Merck Sorono, Biogen, and Genzyme; and receives research support from Novartis and Biogen. SM received honoraria for lecturing, travel expenses for attending meetings, and financial research support from Almirall, Bayer Health Care, Biogen, Diamed, Genzyme, Merck Serono, Novartis, Novo Nordisk, ONO Pharma, Roche, Sanofi-Aventis, and Teva. CG received speaker honoraria and travel expenses for attending meetings from Bayer Health Care, Genzyme, and Novartis Pharma GmbH. HW received compensation for serving on Scientific Advisory Boards/Steering Committees, for Bayer Healthcare, Biogen Idec, Sanofi—Genzyme, Merck Serono, and Novartis. He has received speaker honoraria and travel support from Bayer Vital GmbH, Bayer Schering AG, Biogen, CSL Behring, EMD Serono, Fresenius Medical Care, Genzyme, Merck Serono, Omniamed, Novartis, and Sanofi Aventis. He has received compensation as a consultant from Biogen Idec, Merck Serono, Novartis, Roche, and Sanofi-Genzyme. HW also received research support from Bayer Healthcare, Bayer Vital, Biogen Idec, Merck Serono, Novartis, Sanofi—Genzyme, Sanofi US, and TEVA Pharma as well as the German Ministry for Education and Research (BMBF), Deutsche Forschungsgesellschaft (DFG), Else Kröner Fresenius Foundation, Fresenius Foundation, Hertie Foundation, Merck Serono, Novartis, NRW Ministry of Education and Research, Interdisciplinary Center for Clinical Studies (IZKF) Muenster, RE Children's Foundation. GMzH has received speaker honoraria and compensation for serving on advisory boards for LFB Pharma and Alexion Pharma. GMzH received research support from the Deutsche Forschungsgemeinschaft (DFG, grant number ME4050/4-1), from the Gemeinnützige Hertie Stiftung, from the Innovative Medical Research (IMF) program of the Westfälische Wilhelms-University Münster, and from the Ministerium für Innovation, Wissenschaft und Forschung (MIWF) des Landes Nordrhein-Westfalen. The remaining authors declare that the research was conducted in the absence of any commercial or financial relationships that could be construed as a potential conflict of interest.
