# Supplementary material for: Immune Cell Profiling of the Cerebrospinal Fluid Provides Pathogenetic Insights Into Inflammatory Neuropathies
Source: Front Immunol. 2019 Mar 21;10:515. doi: 10.3389/fimmu.2019.00515 (PMC6448021; doi:10.3389/fimmu.2019.00515)
Supplement: Supplementary file 1 [file Data_Sheet_1.docx]

Supplementary Material

# Supplementary Tables

**Supplementary Table 1**

**GBS vs. IIH**

| **Parameter** | **AUC** |
| --- | --- |
| Protein | 0.95 |
| BBB disruption | 0.89 |
| T cells | 0.75 |
| Plasma cells | 0.73 |
| NK cells | 0.73 |
| HLA-DR+ T cells | 0.70 |
| HLA-DR+CD4+ T cells | 0.70 |
| Non-classical monocytes | 0.69 |
| Intermediate monocytes | 0.68 |
| HLA-DR+CD4+CD8+ T cells | 0.67 |
| Classical monocytes | 0.66 |
| Monocytes | 0.65 |
| HLA-DR+CD8+ cells | 0.65 |
| NKT cells | 0.64 |
| B cells | 0.62 |
| CD4+ T cells | 0.62 |
| CD4/CD8 ratio | 0.60 |
| CD8+ T cells | 0.60 |
| CD56bright NK cells | 0.59 |
| CD4+CD8+ T cells | 0.57 |
| OCBs | 0.54 |
| Lymphocytes | 0.52 |
| Intrathecal Ig synthesis | 0.50 |
| CD56dim NK cells | 0.50 |

**Supplementary Table 2**

**CIDP vs. IIH**

| **Parameter** | **AUC** |
| --- | --- |
| Protein | 0.97 |
| BBB disruption | 0.94 |
| HLA-DR+ T cells | 0.80 |
| HLA-DR+CD4+ T cells | 0.76 |
| CD4+ T cells | 0.75 |
| CD4/CD8 ratio | 0.75 |
| HLA-DR+CD8+ T cells | 0.75 |
| CD8+ T cells | 0.75 |
| T cells | 0.73 |
| NK cells | 0.70 |
| Non-classical monocytes | 0.68 |
| HLA-DR+CD4+CD8+ T cells | 0.67 |
| NKT cells | 0.63 |
| B cells | 0.61 |
| CD4+CD8+ T cells | 0.60 |
| Classical monocytes | 0.59 |
| Intermediate monocytes | 0.58 |
| OCBs | 0.56 |
| Plasma cells | 0.56 |
| Lymphocytes | 0.55 |
| CD56dim NK cells | 0.53 |
| Monocytes | 0.52 |
| Intrathecal Ig synthesis | 0.52 |
| CD56bright NK cells | 0.50 |

**Supplementary Table 3**

**CIDP vs. GBS**

| **Parameter** | **AUC** |
| --- | --- |
| NKT cells | 0.76 |
| Classical monocytes | 0.73 |
| Intermediate monocytes | 0.72 |
| Plasma cells | 0.66 |
| CD4/CD8 ratio | 0.63 |
| CD4+ T cells | 0.63 |
| CD8+ T cells | 0.62 |
| Monocytes | 0.61 |
| CD56bright NK cells | 0.59 |
| HLA-DR+CD8+ T cells | 0.59 |
| NK cells | 0.58 |
| Protein | 0.58 |
| T cells | 0.58 |
| HLA-DR+ T cells | 0.55 |
| Lymphocytes | 0.55 |
| CD56dim NK cells | 0.55 |
| HLA-DR+CD4+CD8+ T cells | 0.54 |
| Non-classical monocytes | 0.53 |
| B cells | 0.52 |
| HLA-DR+CD4+ T cells | 0.51 |
| CD4+CD8+ T cells | 0.49 |
| Intrathecal Ig synthesis | 0.48 |
| OCBs | 0.48 |
| BBB disruption | 0.46 |

# Supplementary Figures

**Supplementary Figure 1**

**
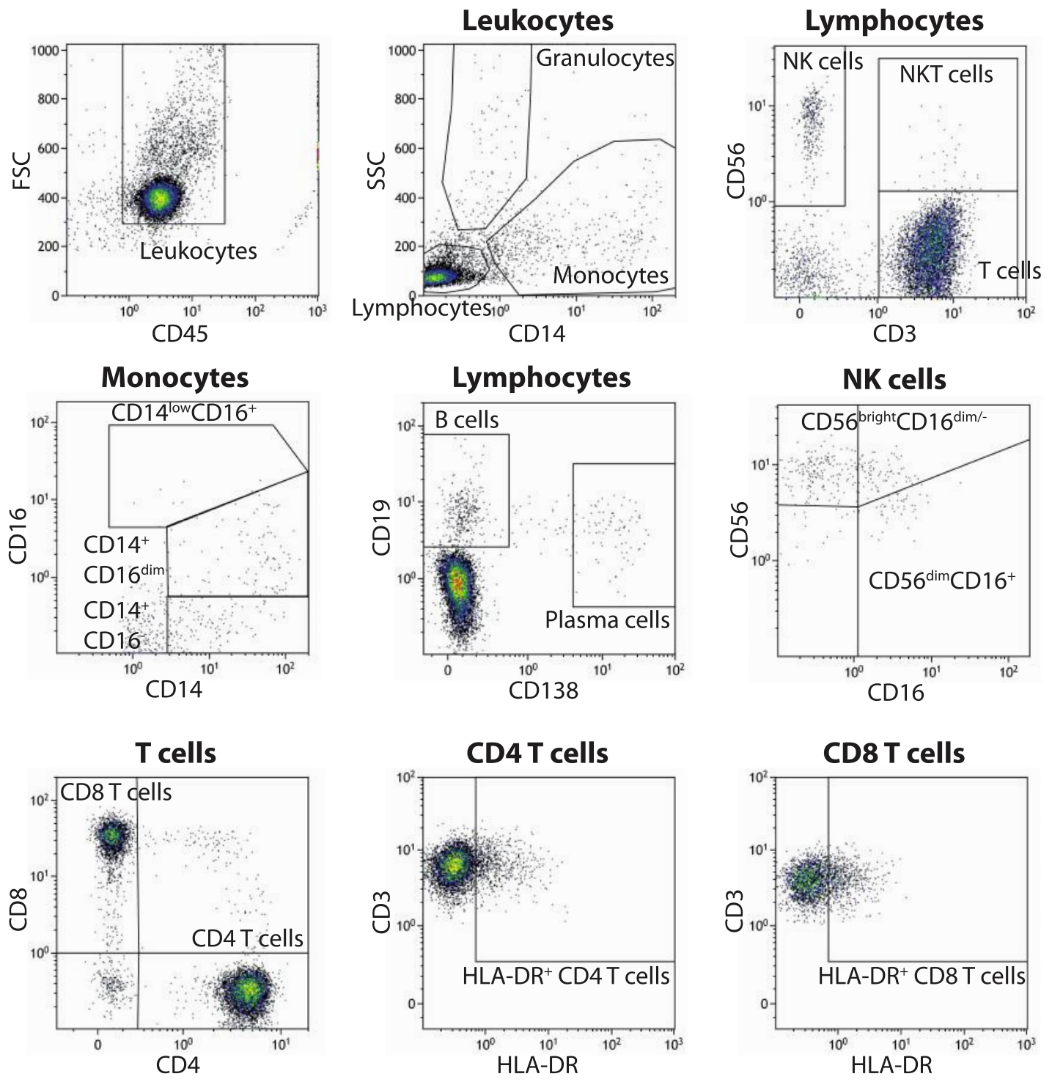
**

**Supplementary Figure 1:** *Gating process of CSF flow cytometry.* Pseudocolor plots depict the gating process. Leucocytes were identified according to forward side scatter (FSC) and CD45 expression. Leukocytes were classified as lymphocytes, granulocytes or monocytes based on CD14 expression and side scatter (SSC). Further gating was performed as indicated.

**
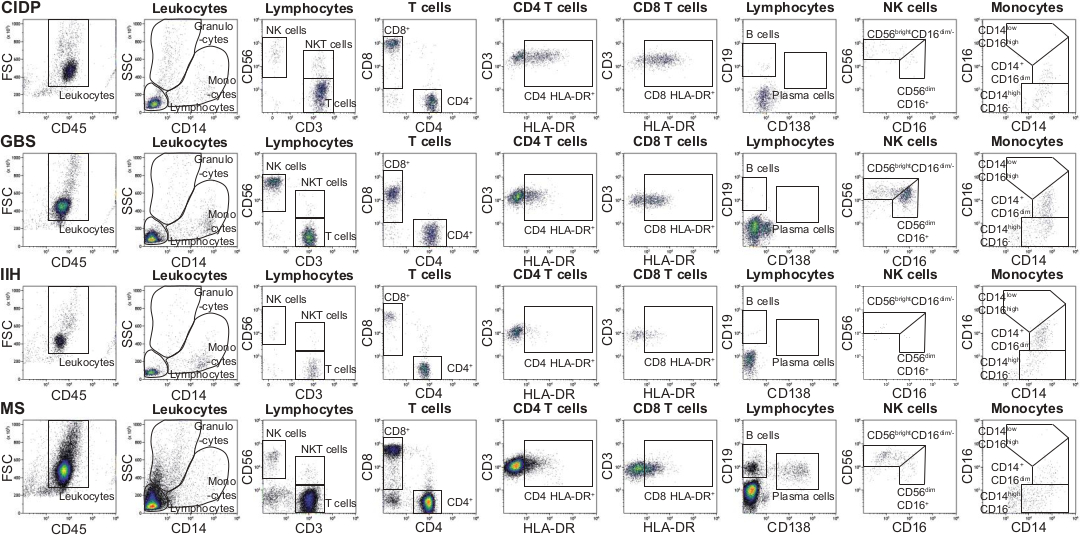
**

**Supplementary Figure 2:** *Representative CSF flow cytometry data.* Pseudocolor plots show representative flow cytometric data of each diagnosis.

**
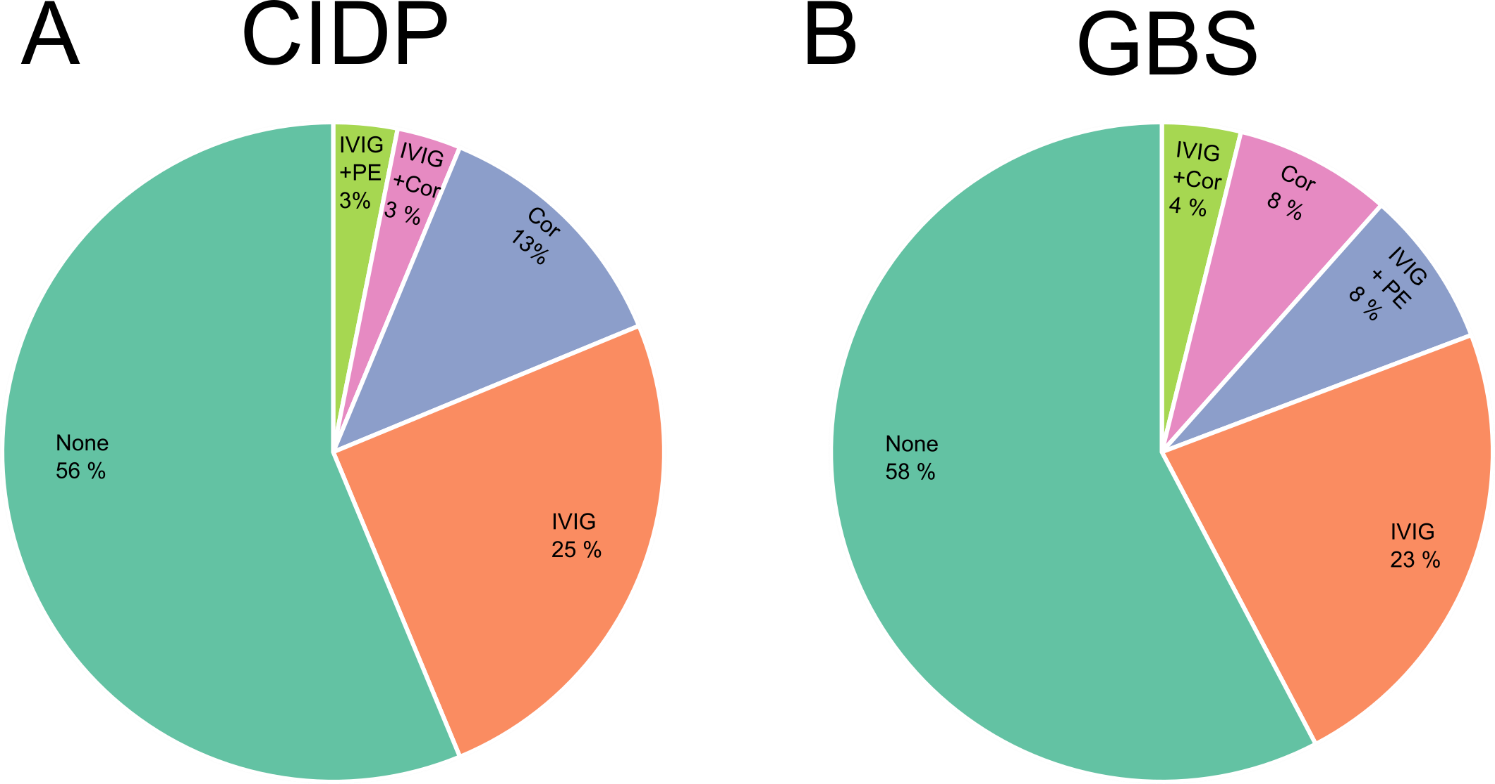
**

**Supplementary Figure 3:** *Treatments of CIDP and GBS patients.* Treatments of the last 3 months of CIDP (A) and GBS (B) patients are plotted in pie charts. Percentages are rounded to nearest integer. CIDP – chronic inflammatory demyelinating neuropathy, Cor – cortisone, GBS – Guillain–Barré syndrome, IVIG – intravenous immunoglobulins, PE – plasma exchange.

**
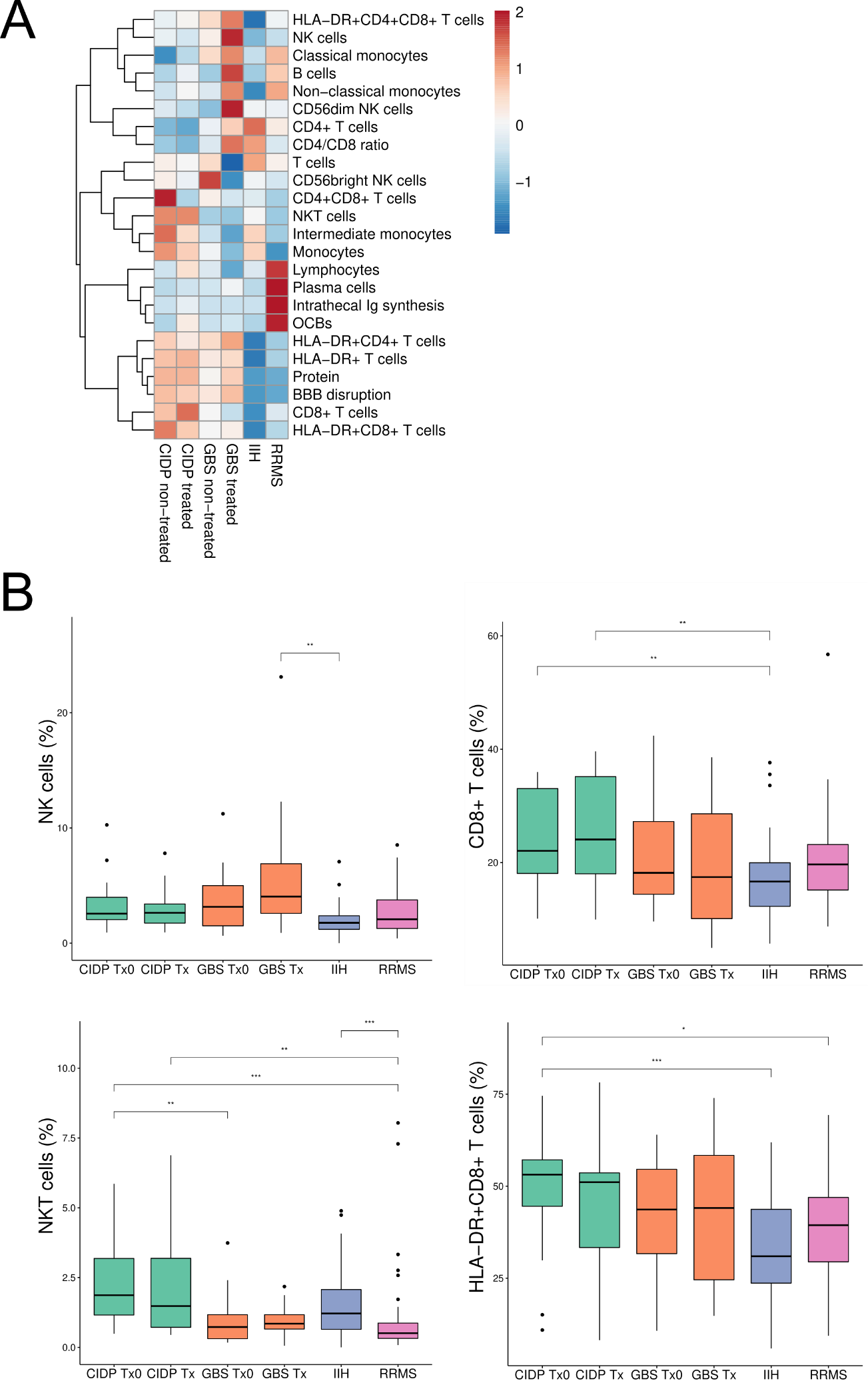
**

**Supplementary Figure 4:** *Immune cell profile in inflammatory neuropathies subdivided by treatments.* (A) The heatmap displays row mean of each CSF parameter per row calculated for chronic inflammatory demyelinating neuropathy (CIDP), Guillain–Barré syndrome (GBS), relapsing-remitting multiple sclerosis (RRMS) and idiopathic intracranial hypertension (IIH). The means were scaled and centered for each row by subtracting the column means from their corresponding column and dividing the columns by their standard deviations. Next, hierarchical clustering was performed with complete linkage method and Euclidean distance measure and visualized in a heatmap. (B) Box plots of selected CSF parameters categorized by diagnosis and subdivided by treatment in the last 3 months. Boxes indicate the lower quartile, median, and upper quartile with whiskers extending to the furthest value within 1.5 times the interquartile range of the box. Outliers are identified individually. The statistical significance of the results was determined using Kruskal-Wallis test and the Dunn test as a post hoc test. Correction for multiple testing was performed by Benjamini-Hochberg's false discovery rate correction. * p < 0.05, ** p < 0.01, *** p < 0.001. BBB – blood-brain barrier, OCBs – oligoclonal bands, Ig – immunoglobulin, Tx – treated, Tx0 – non-treated.


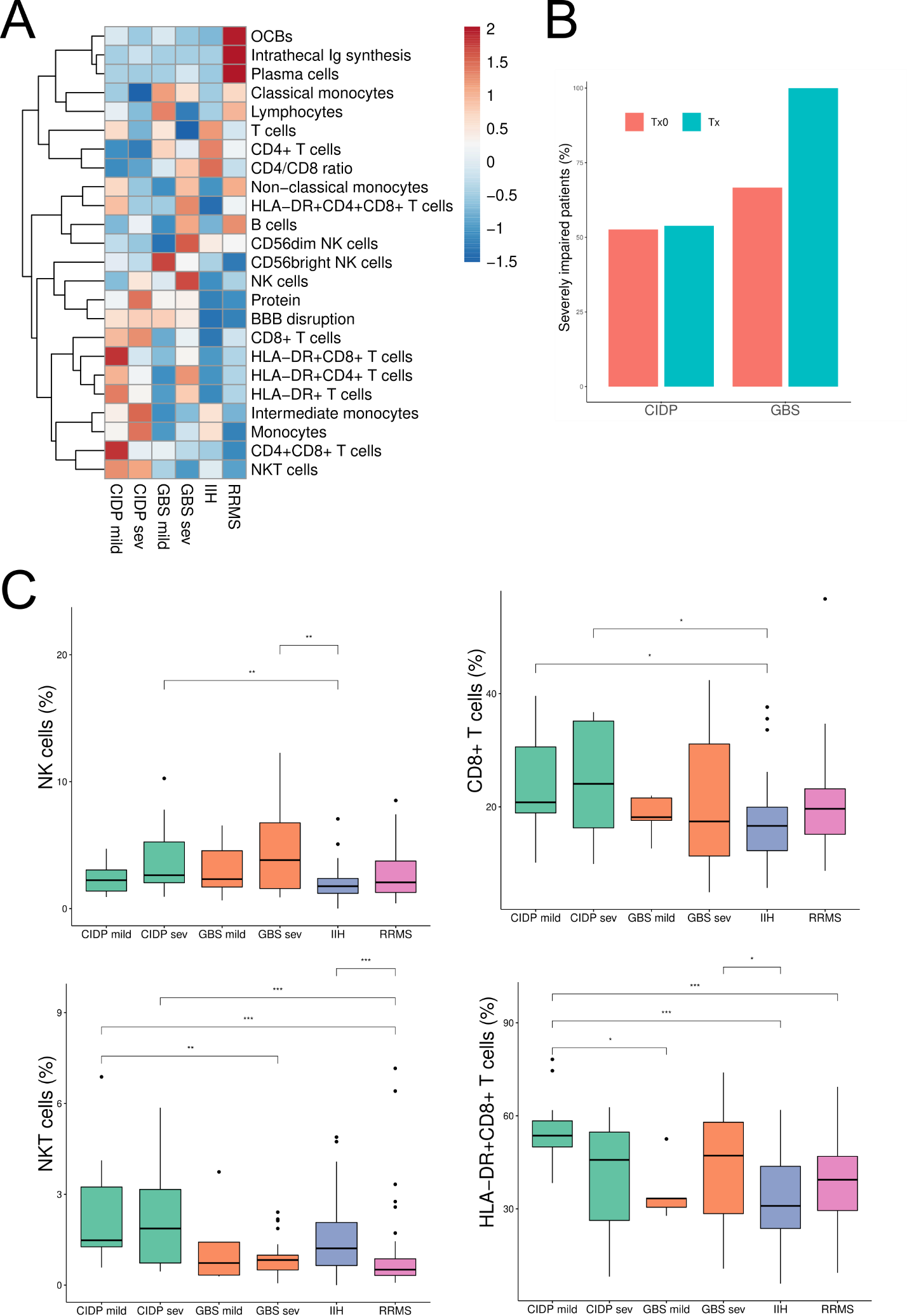


**Supplementary Figure 5:** *Immune cell profile in inflammatory neuropathies subdivided by disease severity.* (A) The heatmap displays row mean of each CSF parameter per row calculated for chronic inflammatory demyelinating neuropathy (CIDP), Guillain–Barré syndrome (GBS), relapsing-remitting multiple sclerosis (RRMS) and idiopathic intracranial hypertension (IIH). The means were scaled and centered for each row by subtracting the column means from their corresponding column and dividing the columns by their standard deviations. Next, hierarchical clustering was performed with complete linkage method and Euclidean distance measure and visualized in a heatmap. (B) Bar plot shows the percentage of severely impaired GBS/CIDP patients based on modified Rankin scale (mRS) subdivided by treatment. Patients with an mRS score of 1 or 2 were classified as mildly impaired, patients with an mRS score of 3 to 5 were classified as severely impaired. (C) Box plots of selected CSF parameters categorized by diagnosis and subdivided by disease severity. Boxes indicate the lower quartile, median, and upper quartile with whiskers extending to the furthest value within 1.5 times the interquartile range of the box. Outliers are identified individually. The statistical significance of the results was determined using Kruskal-Wallis test and the Dunn test as a post hoc test. Correction for multiple testing was performed by Benjamini-Hochberg's false discovery rate correction. * p < 0.05, ** p < 0.01, *** p < 0.001. BBB – blood-brain barrier, OCBs – oligoclonal bands, Ig – immunoglobulin, sev – severe, Tx – treated, Tx0 – non-treated.

**
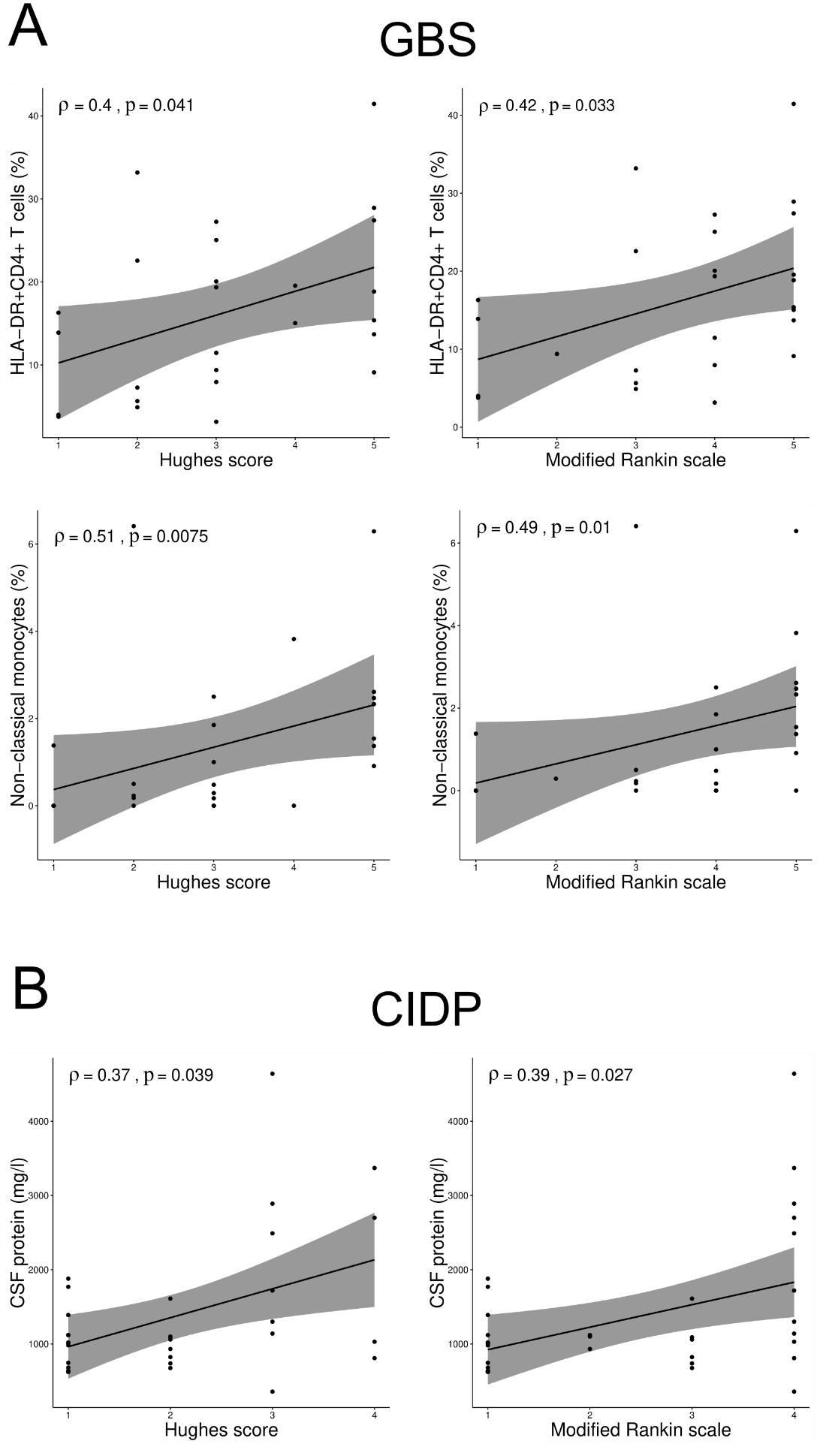
**

**Supplementary Figure 6:** *CSF parameters correlate with disability of immune-mediated neuropathies*. Correlation between Hughes score and modified Rankin scale and CSF parameters were calculated by Spearman’s rank correlation coefficient ρ. Scatterplots are depicted of GBS patients (A) and CIDP patients (B) with a linear regression line and 95% confidence interval (grey area). CIDP – chronic inflammatory demyelinating neuropathy, GBS – Guillain–Barré syndrome.
